# Supplementary figures and images for: IL-10 and class 1 histone deacetylases act synergistically and independently on the secretion of proinflammatory mediators in alveolar macrophages
Source: PLoS One. 2021 Jan 20;16(1):e0245169. doi: 10.1371/journal.pone.0245169 (PMC7816993; doi:10.1371/journal.pone.0245169)

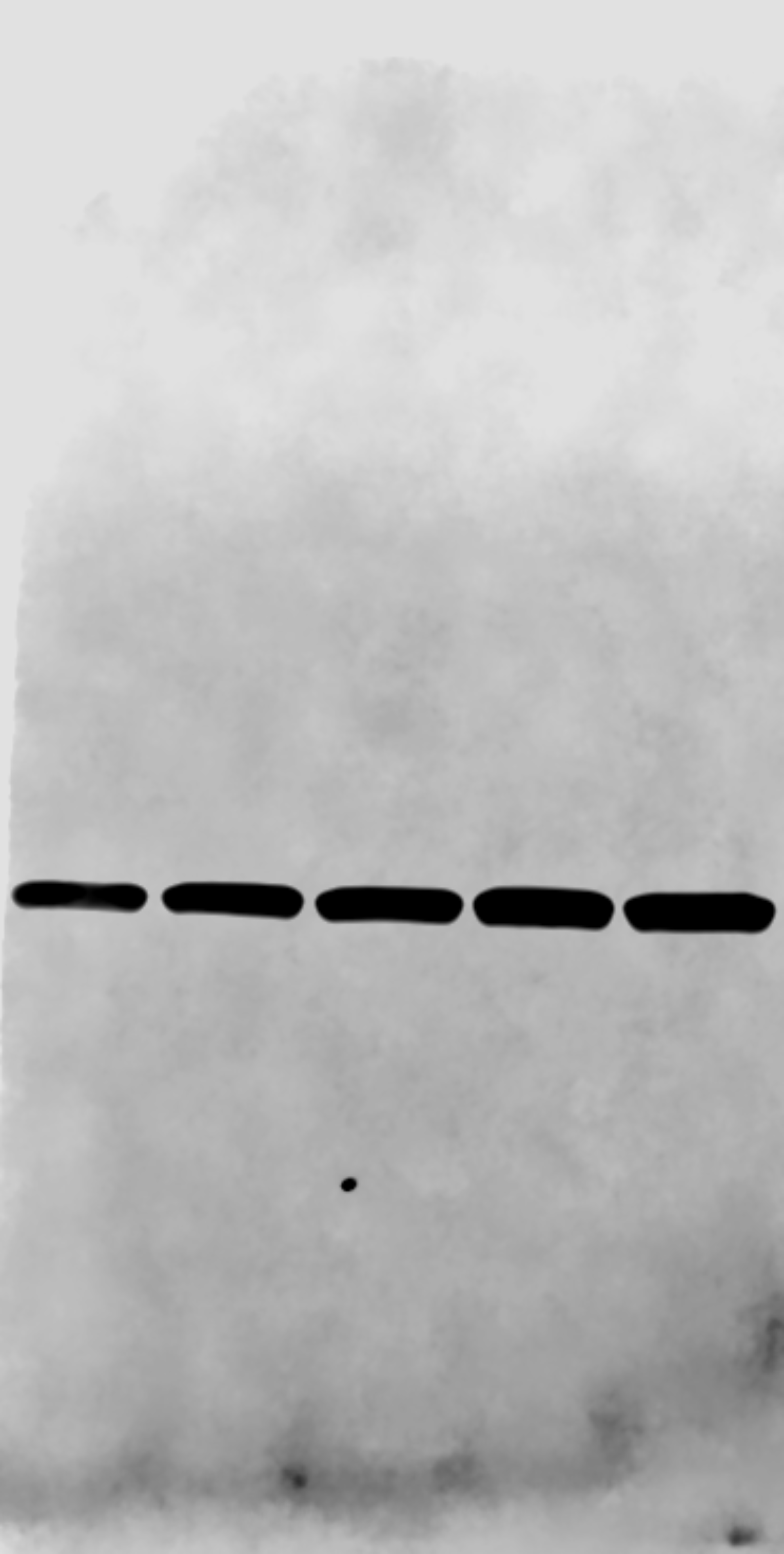

Supplement: S1 File — (ZIP) [file pone.0245169.s001.zip › Raw Blots/GAPDH.tif]

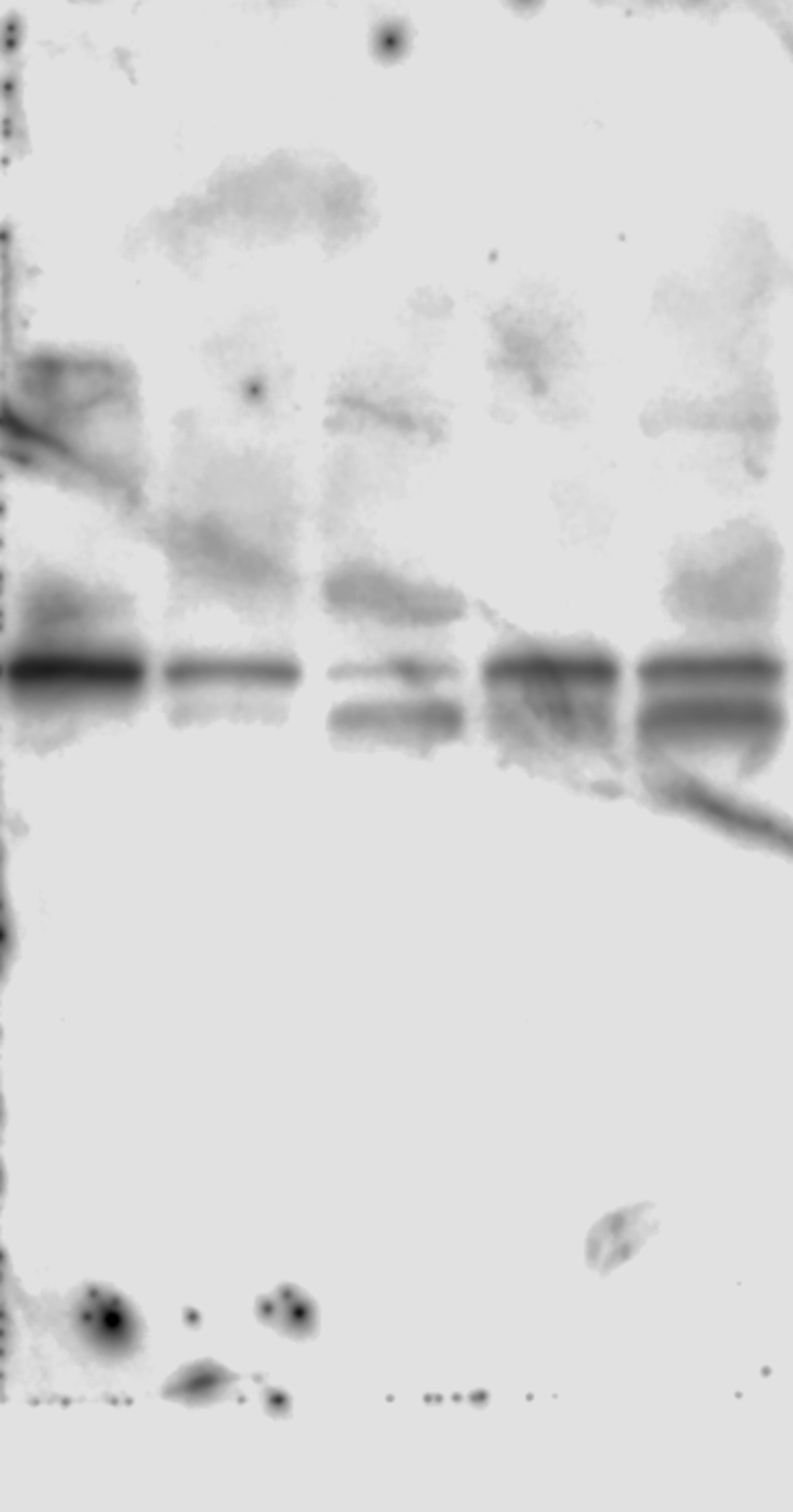

Supplement: S1 File — (ZIP) [file pone.0245169.s001.zip › Raw Blots/IkBa Total.tif]

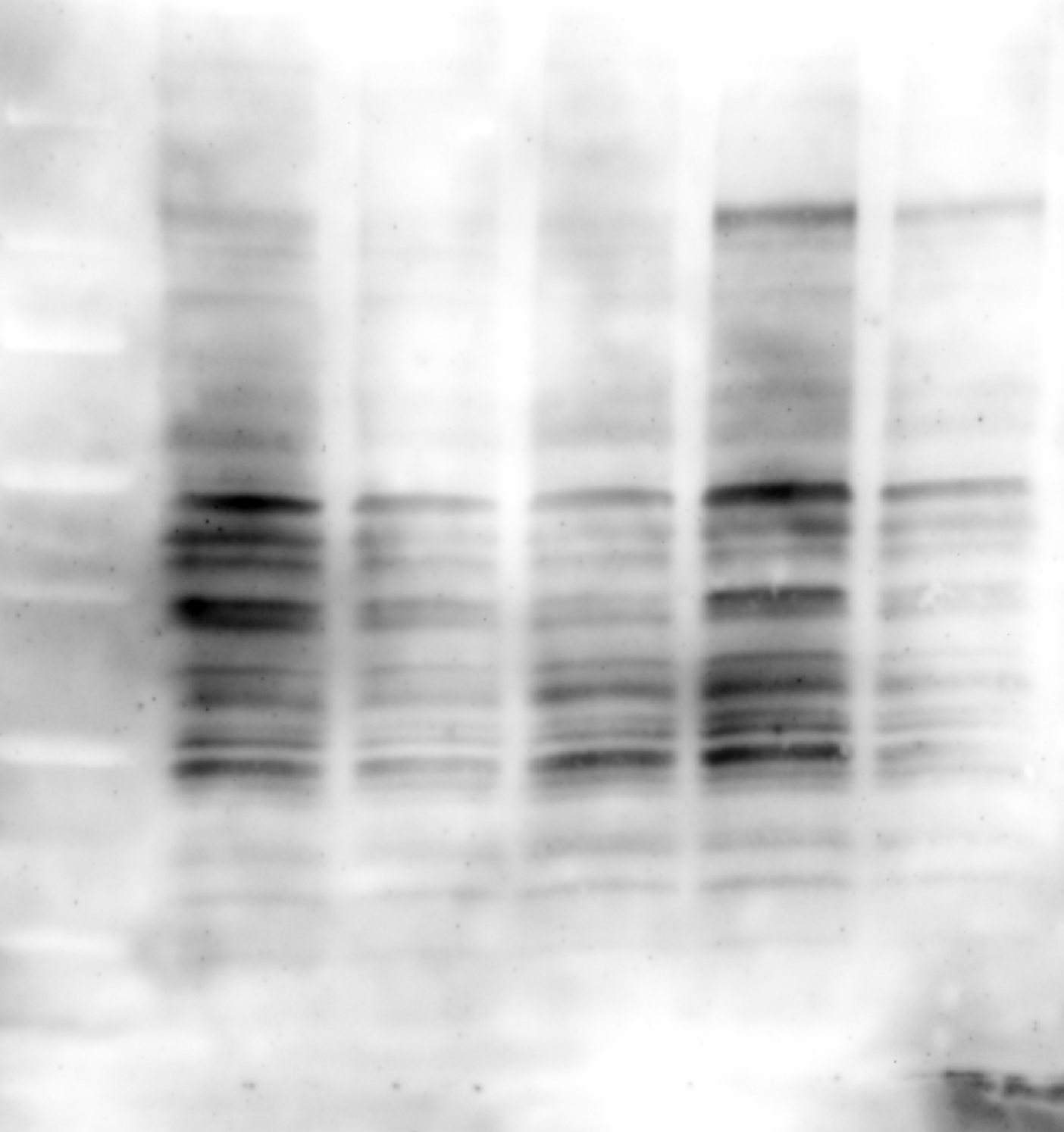

Supplement: S1 File — (ZIP) [file pone.0245169.s001.zip › Raw Blots/P-IkBa.tif]

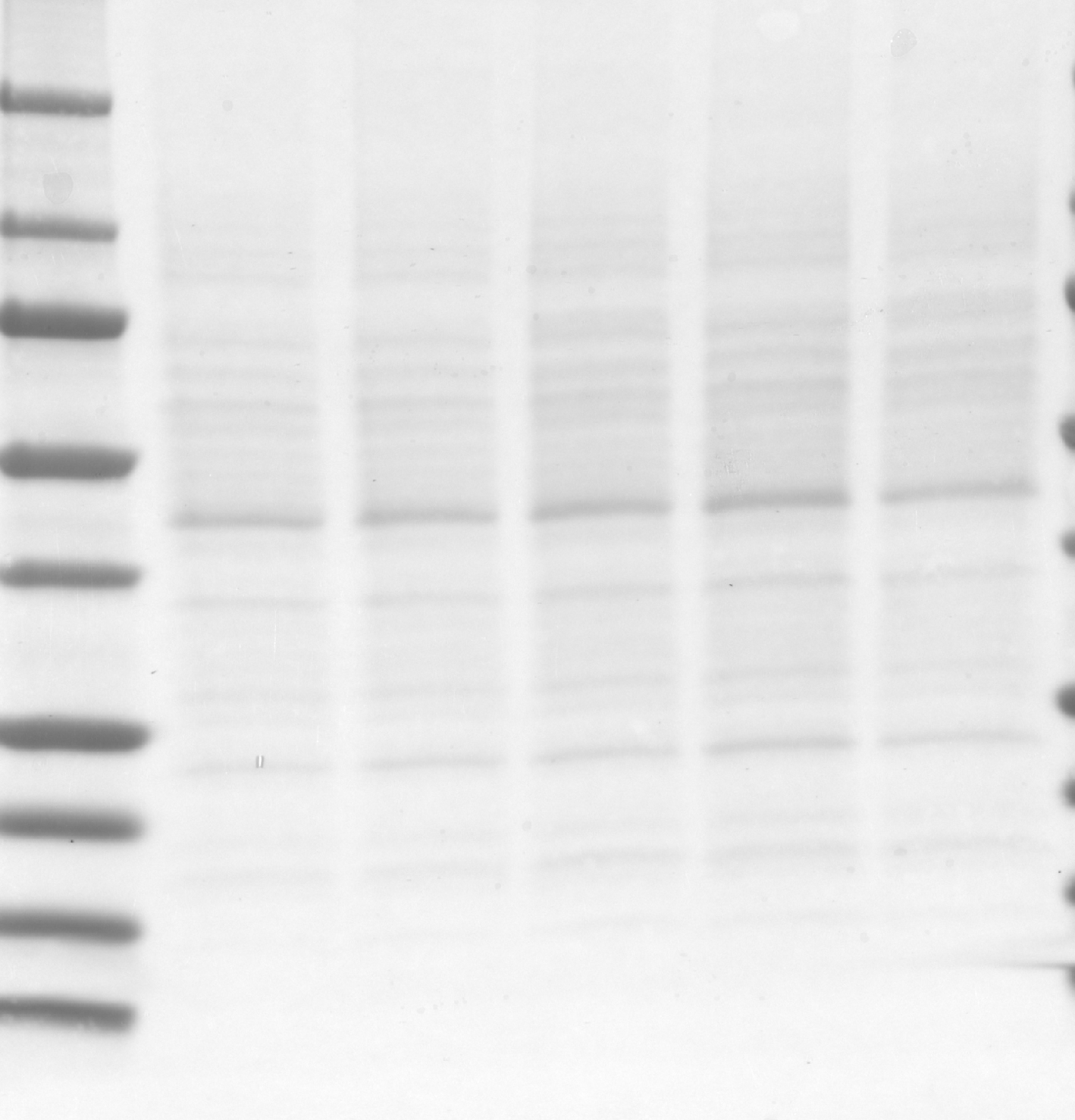

Supplement: S1 File — (ZIP) [file pone.0245169.s001.zip › Raw Blots/Total Protein Ponceau.tif]
