# Supplementary material for: IL-10 and class 1 histone deacetylases act synergistically and independently on the secretion of proinflammatory mediators in alveolar macrophages
Source: PLoS One. 2021 Jan 20;16(1):e0245169. doi: 10.1371/journal.pone.0245169 (PMC7816993; doi:10.1371/journal.pone.0245169)
Supplement: Attachment — Submitted filename: PLOS One - Review Response v3.docx [file pone.0245169.s002.docx]

October 15, 2020

To the PLOS One Editorial Team,

We want to thank the PLOS One Editorial Team for the opportunity to address the issues identified in the peer-review process, and the reviewers for their insightful, constructive feedback. We do believe that the edits made based on the reviewers’ suggestions significantly enhance the quality and impact of our manuscript.

The specific edits made are shown below each review item in red, while corresponding edits in the manuscript can be followed by Tracked Changes.

Reviewer #1: In the present study "IL-10 AND HDAC3-DEPENDENT REGULATION OF TLR SIGNALING AND IL-12b SYNTHESIS IN ALVEOLAR MACROPHAGES" the authors try to discover the effect of IL-10 and HDAC3 on LPS induced alveolar macrophage inflammatory response. Here are some major concerns:

1. The current research utilizes MS-275 to discuss the function of HDAC3. However, it is an inhibitor of both HDAC1 and HDAC3. If the authors insist on claiming the effect of HDAC3, a specific inhibitor of HDAC3, or genetic modification of HDAC3 expression should be used. Judging from the current data, the authors can only make conclusions about the function of HDAC1 and HDAC3, instead of a specific HDAC3 function as claimed in the manuscript.

We appreciate the reviewer’s insightful note. We have edited the manuscript throughout to reflect that MS275 may inhibit all class 1 HDACs (HDAC1, HDAC2, and HDAC3).

2. Western blot in Figure 3 should show both the phosphorylated band and the total protein band. A loading control should be shown as well.

We thank the reviewer for the comment and have added the S32/36 P-IκBα and GAPDH blots to the figure (Figure 4A)

3. The authors need to analyze the nuclear translocation of p65 and p50 to claim a regulation of NF-kappaB. Phosphorylation of IkappaBs and the expression level of signal molecules of this pathway are not sufficient to indicate its activation.

We thank the reviewer for the comment, and have added Figure 5 to the manuscript detailing the change in nuclear RelA with IL-10 and Class 1 HDAC inhibition under stimulation with LPS.

4. There is not any evidence about the upper/lower stream relationship of IL-10 and HDAC3. Because AS101 and MS-275 have combinatorial effect in many results, it is more likely IL-10 and HDAC3 go through independent mechanisms. The authors need to correct the manuscript and Figure 7.

We thank the reviewer for the comment and have changed Figure 7 (now Figure 8a) to specifically define AS101 as inhibiting IκBα phosphorylation, while MS275 specifically targets P-IκBα ubiquitination/degradation.

5. In figure 1A, IL-12b has the highest mRNA expression when LPS is combined with AS-101 and MS-275. However, in the same condition IL-12b supernatant concentration is quite low as shown in Figure 5B. The authors need to explain the discrepancy.

We thank the reviewer for the note. Based on this, we added an explanation of the mechanism of action AS101 has on cytokine secretion, based on previously published data. “AS101 is known to disrupt IL-10 secretion by inhibiting integrin activity (1). The loss of integrin activity likely explains the discrepancy observed between the transcriptional and secreted cytokine analysis.”

6. In figure 1C, why would AS-101 increase the mRNA expression of HDAC3? Does it mean

We thank the reviewer for the comment, and have removed the HDAC3 expression analysis from the paper. MS275 inhibits class 1 HDACs (HDAC1, HDAC2, and HDAC3) independent of their transcriptional regulation. Disruption of class 1 HDAC function has broad effects on the transcriptional regulation of many genes, as evident by figures 1-4, and the mechanism of action of MS275 had been described in the first paragraph of the Methods section: “Agent MS275, a selective class 1 HDAC inhibitor (2) (Entinostat, Santa Cruz Biotechnology, sc-279455), was reconstituted to a stock concentration of 10 mM in DMSO.”

7. The authors need to discuss why TNF-alpha, MIF, IL-6 and CXCL2 show different pattern than IL-12b, as they are supposed to be regulated by NF-kappaB too.

We thank the reviewer for the comment and acknowledge the importance of NF-κB in the expression of TNF-alpha, MIF, IL-6, CXCL2, and IL-12b. As a potent inducer of nuclear NF-κB, LPS did induce the secretion of TNF-alpha, IL-6, CXCL2, IL-10, and IL-12b over base line concentrations, however the mechanism by which IL-10 and class 1 HDACs modulate the secretion of these cytokines is a topic to be investigated in future work. Macrophage MIF secretion is known to be dose-dependent after LPS stimulation, (3) suggesting a more complex regulatory mechanism beyond merely NF-κB, and this regulation is beyond the scope of the current project. The following has been added to the Results section, paragraph 10: “Macrophage MIF secretion is known to be dose-dependent after LPS stimulation, (3) suggesting a more complex regulatory mechanism beyond NF-κB, that is beyond the scope of the current project.”

8. The authors need to discuss why alveolar macrophages show different pattern than other macrophages in their cytokine/chemokine expression upon stimulation with LPS, especially the lack of IL-10 induction.

We thank the reviewer for the comment, and apologize for the confusion. LPS did induce IL-10 secretion over baseline levels. To better show this, we have added the statistical comparisons between the vehicle (DMSO) treatment group and the other 4 groups in the study (Figure 6A).

Reviewer #2: The manuscript entitled “IL-10 and HDAC3 dependent regulation of TLR signaling and IL-12b synthesis in alveolar macrophages” demonstrated that alveolar macrophage (AM) upregulates IL-10 and IL-12b production with a high HDAC3 dependent manner. Therefore, the authors claimed that HDAC3 might be a potential target for macrophage-initiated pulmonary inflammation in acute lung injury. Throughout this article, the authors clearly depicted the balance between pro-inflammatory and anti-inflammatory cytokine production after LPS treatment in vitro. In addition, the authors utilized inhibitions of IL-10 and HDAC to alveolar macrophage to support the hypothesis. Currently, the acute respiratory distress syndrome (ARDS) has become an important clinical challenge and we need to understand the pathogenesis of this disease. The article will be helpful to understand the underlying mechanisms of ARDS. However, several concerns must be addressed for better quality.

1. Acute Respiratory Distress syndrome (ARDS) can be induced by a variety immunological responses and the activation of TLR. According to previous reports (S Han and RK Mallampalli, 2015), ARDS induces both TLR2 (microbial cell wall components) and TLR4 (LPS) as a consequence of bacterial mediated sepsis. The authors only focused on TLR4 in this article. Please provide more rationale to choose TLR4 and support idea the involvement of TLR2 in Acute lung injury.

We thank the reviewer for the comment and have added the above mentioned reference to the manuscript. Also, we went on the describe our rational for choosing TLR4 over TLR2 in our investigation by including references describing the importance of TLR4 activation to the development of ARDS and the dual signaling mechanism (MyD88 Dependent and Independent) nature of TLR4 unique to TLR4. The following has been added to the first paragraph of the Discussion section: “Recent work has described the activation of both TLR2 (recognition of bacterial cell wall components) and TLR4 (recognition of lipopolysaccharide) as a consequence of bacterial mediated sepsis leading to Acute Respiratory Distress Syndrome (ARDS) (4, 5). However TLR4 dependent inflammatory responses have been shown to be essential to the development of ARDS in murine models (6) and in contrast to TLR2, only activation of TLR4 is associated with the activation of alveolar macrophages in human lungs (7). This interaction leads to intracellular signaling mediated chiefly through two adaptor protein systems, the Myeloid Differentiation Response Protein 88 (MyD88) and TIR-domain-containing adapter-inducing interferon-β (TRIF). TLR4 is unique among TLRs as it can signal via both MyD88 dependent and independent (TRIF dependent) pathways (8).”

2. The authors used alveolar macrophage cells (MH-S) in this study. However, it is very difficult to make a conclusion only with in vitro system. Although bacterial pneumonia is the most common cause of ARDS, there are a variety of intercellular communications to induce ARDS. It is unlikely to demonstrate the pathogenesis of ARDS via only the involvement of alveolar macrophage cells. Please consider using animal model for in-depth understanding. For example, LPS injection to the animal model to mimic ARDS with AS101/MS275 treatment.

We thank the reviewer for the comment, and indeed plan to conduct detailed animal studies on the role of class 1 HDACs in animal models of ARDS. Animal experiments are beyond the scope of the current project. In addition, generation of knockout mice, as requested, was not feasible within the timeframe of the response to review, given our ability to only run our lab part time, due to institutional Covid-related restrictions. We include a detailed description of the planned animal work in paragraph 6 of the Discussion section: “Future work to address the role of specific class 1 HDACs (HDAC1, HDAC2, and or HDAC3) in the pathogenesis of ARDS *in-vivo* will involve the use of conditional knockout mice, as loss of these genes has been demonstrated to be embryonically lethal (9, 10). Similarly, constitutive tissue-specific loss of these genes would lead to developmental abnormalities at best. Additionally, more specific pharmacological inhibition (HDAC1: Valproic Acid, HDAC2: Romidepsin, HDAC3: RGFP966) will be utilized to more specifically inhibit these enzymes.”

3. Related with previous suggestion, using IL-10 KO mouse or shIL-10 Knock down mice would be recommended to understand this pathogenesis.

We thank the reviewer for the suggestion. Please see response to item #3. We do plan on integrating IL-10 knockout mice on our upcoming experiments.

4. There was no data about TLR4 activity or TLR4 changes with LPS stimulation. It should be added.

We thank the reviewer for the comment. TLR4 activation with LPS stimulation in macrophages is a well described phenomenon that has been described extensively in the literature. The following has been added to the 1^st^ paragraph of the Results section: “LPS stimulation has been extensively shown to rapidly induce TLR4 activation in macrophages (11-14) and previous work has described the downstream regulatory role IL-10 plays on the expression of IL-12b through the activation of HDAC3 (a class 1 HDAC) in colonic macrophages (15).”

5. In line139, “AS101 inhibited IL-10 protein synthesis”, however, there is no AS101 treated group data to compare with vehicle treated group. Once again, there were no data “AS-101 only treated” and “MS275 only treated” group in most graphs. Please add those data, too. For instance, down-regulation of IL-10/HDAC3 mRNA level must be pre-qualified after inhibitory chemical treatment (whether IL-10 inhibitor and HDAC3 inhibitor work well or not).

We thank the reviewer for the comment and have edited the manuscript throughout to reflect that the changes observed with AS101 and MS275 treatment are merely in the context of LPS stimulation.

6. Please put the statistics between the DMSO and DMSO+LPS groups in figures.

We thank the reviewer for the suggestion, and based on it, we have added the requested statistics to all the figures.

7. In figure legends, there are many repeats in each legend. Please make them more simplify.

We thank the reviewer for the comment. We have simplified the legends across all Figures.

8. In Figure 4, there is no house-keeping protein in Western blot results.

We thank the reviewer for the comment. We added the S32/36 P- IκBα and GAPDH blots to the figure (Figure 4A).

9. In Figure 5c and Figure 6B (AS101+LPS), Please re-identify the statistical analyses. It is confusing.

We apologize for the confusion, and thank the reviewer for the suggestion. We re-identified the statistical analysis in the requested graphs, now renamed Figure 6C and 7B.
