# Supplementary material for: IL-10 and class 1 histone deacetylases act synergistically and independently on the secretion of proinflammatory mediators in alveolar macrophages
Source: PLoS One. 2021 Jan 20;16(1):e0245169. doi: 10.1371/journal.pone.0245169 (PMC7816993; doi:10.1371/journal.pone.0245169)
Supplement: Attachment — Submitted filename: Response_to_Reviewer_3.docx [file pone.0245169.s003.docx]

Dear Editor,

We appreciate the Reviewer’s additional insightful comments and constructive feedback, along with the opportunity to further edit our manuscript.

Please see our response below.

Reviewer #1: The current study "IL-10 AND CLASS 1 HISTONE DEACETYLASES ACT SYNERGISTICALLY AND INDEPENDENTLY ON THE SECRETION OF PROINFLAMMATORY MEDIATORS IN ALVEOLAR MACROPHAGES" made a lot of improvement compared with the previous version. Here are some more minor concerns to be addressed:

1. In figure 5, the DMSO + LPS group did not show increase of RelA nuclear localization compared with DMSO group. This group sets the baseline of the current experiment. If the authors believe it is because of a too late time point, an earlier time point should be selected for this experiment.

As our western blot demonstrated (Figure 4), MH-S cells appear to maintain a relatively high level of Phospho-IκBα in the unstimulated control group. Though IκBα is not readily degraded with this treatment, constitutive phosphorylation of IκBα could result in a steady state of nuclear NFκB activity difficult to overcome with LPS treatment. It is also possible that at the time point we investigated these treatments RelA was already exported to the cytoplasm. However, our key finding and focus here is that treatment with either MS275 or AS101 significantly reduced nuclear RelA localization.

2. The authors claim AS101 reduces phosphorylation of IkBa. In such case, the subsequent degradation should be low and the total protein of IkBa should be high. In figure 4, the IkBa total protein is low in the AS101 group. Please explain. Also, how is this IL-10 synthesis inhibitor related to IkBa phosphorylation?

AS101 was applied at the time of LPS stimulation and may not have had time to interfere with the degradation of IκBα. Since Phospho- IκBα is constitutively high in these cells additional kinases (Other than IkKs) may be involved in this phenotype. This was not focus of this investigation but is definitely an interesting topic we plan to pursue in future work. In the discussion of figure 4 we state that it is likely that only newly synthesized IκBα is unphosphorylated and with constitutively high IκBα phosphorylation in MH-S cells additional regulatory steps are likely what contributes to the inhibition of IκBα degradation.

3. The authors addressed MYD88 dependent and independent pathways. However, the current study only detected the expression of the components of these pathways, which is not sufficient to indicate activity. The authors may do some discussions according to the current data, but putting these pathways in the conclusion is too stretchy.

We have edited our discussion to note that AS101 and MS275 act on the transcription of MyD88 Dependent and independent pathway components. Lines 453-471:

“We also demonstrate that IL-10 and class 1 HDACs both independently and synergistically regulate IL-12b transcription, and that IL-10 modulates the TLR -NF-κB signaling pathway by suppressing the transcription of both MyD88-dependent and MyD88-independent pathways. The latter finding provides an additional means of IL-10 modulating its own transcription and secretion (**Figure 1A and 6A)**, in addition to regulating its own receptor (52), or post-transcriptionally (53-55), and corroborates the finding by He et al that TLR4-NF-κB signaling differentially regulates IL-10 and IL-12b transcription, through regulation of c-fos synthesis and differential binding of the NF-κB transcription factor to the two cytokine promoters. AS101 is known to disrupt IL-10 secretion by inhibiting integrin activity (64). The loss of integrin activity likely explains the discrepancy observed between the transcriptional and secreted cytokine analysis. In addition, we showed that IL-10 and class 1 HDAC inhibition differentially affected IκBα synthesis and proteolytic degradation, shifting the TLR-NF-κB signaling to anti-inflammatory territory. Both enhance IkKα and RelA expression, but only IL-10 inhibition increases p50 gene expression. Lastly, we show that IL-10 inhibited the transcription of both MyD88-dependent and MyD88-independent pathway components, while class 1 HDAC inhibition only appears to affect the transcription of MyD88-dependent components. **Figure 8A-B** summarizes the effect of IL-10 and class 1 HDAC inhibition on the modulation of IκBα phosphorylation (AS101) and ubiquitination/degradation (MS275) in the TLR4 signaling pathway, and the proposed interaction between IL-10, IL-12b and class 1 HDACs with LPS stimulation.”

4. The authors need to explain IL-10 mRNA expression is not increased by LPS in figure 1A but protein is increased in figure 6A.

It is likely that IL-10 synthesis is regulated post-transcriptionally through a negative feedback loop mechanism, and we are planning a new project to study this specific topic in the very near future. The following was added to lines 315- 320:

“Of note, IL-10 mRNA transcription was noted to remain unchanged with LPS stimulation (Figure 1A), while protein synthesis increased (Figure 6A. It appears that anti-inflammatory IL-10 transcription takes place constitutively with an inhibitory process limiting protein secretion, and stimulation with LPS enables removes this inhibitory step to allow for greater protein synthesis. The topic of post-transcriptional IL-10 regulation is to be examined in a separate project.”

Sincerely,

The author team.
